# LncRNA BC200/miR-150-5p/MYB positive feedback loop promotes the malignant proliferation of myelodysplastic syndrome

**DOI:** 10.1038/s41419-022-04578-2

**Published:** 2022-02-08

**Authors:** Zhaoping Liu, Pan Wang, Shunling Yuan, Yanyan Wang, Pengfei Cao, Feng Wen, Hui Li, Lin Zhu, Long Liang, Zi Wang, Bin Hu, Fuxiang Zheng, Jing Liu, Xiaojuan Xiao, Ji Zhang

**Affiliations:** 1 Department of Clinical Laboratory, Shenzhen Traditional Chinese Medicine Hospital, Shenzhen, 518033 Guangdong, China; 2 The First Affiliated Hospital, Department of Clinical Laboratory, Hengyang Medical School, University of South China, Hengyang, 421001 Hunan China; 3 Molecular Biology Research Center & Center for Medical Genetics, School of Life Sciences, Central South University, Changsha, 410078 Hunan China; 4 Department of Hematology, Xiangya Hospital, Central South University, Changsha, 410008 China; 5 The First Affiliated Hospital, Department of Hematology, Hengyang Medical School, University of South China, Hengyang, 421001 Hunan China; 6 Hunan Province Key Laboratory of Basic and Applied Hematology, Central South University, Changsha, 410078 Hunan China

**Keywords:** Haematological cancer, Myelodysplastic syndrome

## Abstract

Myelodysplastic syndrome (MDS) is a group of heterogeneous hematologic malignancies with a risk of transformation to acute myeloid leukemia. Understanding the molecular mechanisms of the specific roles of long noncoding RNAs (lncRNAs) in MDS would create novel ways to identify diagnostic and therapeutic targets. The lncRNA BC200 is upregulated and acts as an oncogene in various cancers; however, its expression, clinical significance, and roles in MDS remain unclear. Here, we found that BC200 was highly expressed in MDS patients compared with normal individuals. Knockdown of BC200 inhibited MDS cell proliferation, colony formation, and cell cycle progression in vitro and suppressed the growth and invasiveness of MDS cells in vivo. Mechanistic investigations revealed that BC200 functioned as a miRNA sponge to positively regulate the expression of MYB through sponging miR-150-5p and subsequently promoted malignant proliferation of MDS cells. Conversely, we found that BC200 was a direct transcriptional target of MYB, and knockdown of MYB abolished the oncogenic effect of BC200/miR-150-5p. Taken together, our results revealed that the BC200/miR-150-5p/MYB positive feedback loop promoted the proliferation of MDS cells and is expected to be a potential biomarker and therapeutic target in MDS.

## Introduction

Myelodysplastic syndrome (MDS) is a clonal hematological malignancy characterized by ineffective hematopoiesis, progressive cytopenia and clonal evolution to acute myeloid leukemia [1, 2]. The pathophysiology of MDS is a multistep process involving cytogenetic changes, gene mutations, or both. MDS frequently has unbalanced translocations and deletions, which implicates the loss of tumor suppressor gene function or the activation of oncogenes [3]. Malignant clonal hematopoietic cells often coexist and compete with normal hematopoietic cells for a considerable period of time in the bone marrow (BM) of MDS patients. When malignant clonal cells become dominant in BM, the disease progresses to AML [4]. The current treatment approaches include hypomethylation chemotherapy and immunomodulatory drugs. Patients with MDS are usually at an advanced age and may have comorbidities that make them ineligible for these treatment options due to excessive toxicity [5]. Thus, better understanding of the pathogenesis of MDS is crucial for developing novel and effective treatment strategies against this fatal disease.

Increasing evidence has shown that long noncoding RNAs (lncRNAs) play crucial roles in contributing to the pathogenesis and progression of various tumors [6, 7]. LncRNAs are a class of transcripts that are >200 nucleotides and do not contain any extended open reading frames [8]. LncRNAs participate in the regulation of target gene expression through various mechanisms, and the most common regulatory approach for lncRNA regulating its target gene is to act as microRNA (miRNA) sponges [9]. In the lncRNA-miRNA-mRNA regulatory network, lncRNA acts as competing endogenous RNAs (ceRNAs) of specific mRNAs [10]. Numerous studies have shown that lncRNAs are frequently dysregulated in various cancers and are involved in a wide range of biological processes, such as malignant proliferation, migration, invasion, and metastasis [11, 12]. The aberrant expression signatures of lncRNAs have been revealed, which provides new insights into the biology of MDS. For instance, lncRNA HOXB-AS3 was found to be upregulated in MDS and was associated with poor prognosis [13]. Moreover, deregulation of several lncRNAs, such as H19, LEF1-AS1WT1-AS, and TCL6, is associated with higher-risk MDS [14]. However, the biological mechanisms of numerous lncRNAs in the pathogenesis and progression of MDS need to be further explored.

Recently, a growing number of studies have revealed that BC200, also known as brain cytoplasmic RNA 1 (BCYRN1), is elevated in a variety of cancer cells, including lung, breast, colorectal, gastric, ovary, and cervix [15–18]. Oncogenic BC200 plays an important role in the proliferation, apoptosis, migration, and invasion of cancer cells [15, 16, 19]. Furthermore, the expression of BC200 is regulated by several factors, including estrogen receptor-α and c-myc. Estrogen induced BC200 expression, which promotes the growth and tumorsphere formation of hepatocellular carcinoma [20]. In non-small-cell lung cancer, BC200 is activated by the transcription factor c-myc, which is critical for cell migration and invasion [21]. However, how BC200 is transcriptionally regulated in MDS and whether it is involved in the pathogenesis and development of MDS are still unclear.

In this study, oncogenic BC200 promoted the growth of MDS cells in vitro and in vivo. Mechanistically, BC200 acted as a sponge for miR-150-5p to upregulate the expression and activity of MYB. Interestingly, MYB, a crucial transcription factor, could directly bind to the BC200 promoter region and induce BC200 transcription, which in turn increased MYB expression. Furthermore, the high expression of BC200 and MYB was significantly negatively correlated with miR-150-5p in MDS patients and MDS cell lines. Thus, our results indicated that the BC200/miR-150-5p/MYB positive feedback loop contributed to the malignant proliferation of MDS cells, providing novel insight into the growth mechanism of MDS and identifying promising therapeutic targets for MDS treatment.

## Materials and methods

### Cell lines and MDS samples

Human MDS cell lines (SKM-1 and MDS-L) and HEK293T cell lines were purchased from ATCC (Manassas, VA, USA). SKM-1 cells were maintained in RPMI-1640 (Gibco, Rockville, MD, USA). MDS-L cells were cultured in RPMI-1640 medium plus 50 μM 2-mercaptoethanol and 100 U/ml IL-3. HEK293T cells were cultured in Dulbecco’s modified Eagle’s medium (Gibco). All media were supplemented with 10% fetal bovine serum (Gibco) and 1% penicillin–streptomycin (Gibco). All cells were cultured at 37 °C in a 5% CO_2_ atmosphere. The cells were authenticated using STR profiles. All cells were routinely tested as mycoplasma-free. All clinical samples were obtained with informed consent at the First Affiliated Hospital of University of South China (Hengyang, China) and Xiangya Hospital of Central South University (Changsha, China). Sample collection was approved by the Hospital’s Protection of Human Subjects Committee. MDS primary samples were cultured in RPMI-1640 medium containing 20% fetal bovine serum and 100 U/ml IL-3.

### Plasmids and transfection

The miR-150-5p inhibitor and inhibitor control, miR-150-5p mimics and miRNA mimics control, small interfering RNAs (siRNAs) targeting BC200 and MYB, and an unrelated sequence used as a negative control siRNA were constructed by RiboBio (Guangzhou, China). The sequences of the reagents were listed in Supplementary Table S1. The cDNA expression plasmids pCMV3-BC200, pCMV3-MYB, and pCMV3-control vector were purchased from Sino Biological (Beijing, China). For the generation of stable cell lines, BC200-specific short hairpin RNA (shRNA) or control vector (GenePharma, Shanghai, China) were transfected into MDS cells, and the cells were screened with 1 μg/ml puromycin for 3–4 weeks after transfection for 48 h.

For transfection, cells in the logarithmic growth phase were inoculated into 12-well plates at 2.5 × 10^5^ cells/well. Transfection was conducted with Ribo FECTTM CP reagent (RiboBio) according to the manufacturer’s instructions in RPMI-1640 culture medium without serum and antibiotic. Six hours after transfection, we continued to culture the cells in the medium containing serum for at least 48 h before the subsequent experiments. The transfection efficiency was determined by quantitative real-time PCR (qRT-PCR).

### RNA extraction and qRT-PCR

Total RNA was isolated with TRIzol reagent (Invitrogen, Carlsbad, CA, USA) and cDNA was generated using a RevertAid First Strand cDNA Synthesis Kit (Thermo Fisher Scientific, Waltham, MA, USA) according to manufacturer’s guidelines. The reverse transcription reaction for miR-150-5p was performed using a Maxima H Minus First Strand cDNA Synthesis Kit (Thermo Fisher Scientific). qRT-PCR was performed using ChamQ^TM^ Universal SYBR^®^ qPCR Master Mix (Vazyme Biotech, Nanjing, China). LncRNA and mRNA expression were normalized to GAPDH expression, and miRNA was normalized to U6 snoRNA. The sequences of the primers used are listed in Supplementary Table S2.

### CCK-8, Ki-67, Edu, and colony formation assays

Transfected cells were seeded into a 96-well plate at a density of 5 × 10^4^ cells/ml. Subsequently,10 μl of Cell Counting Kit-8 (CCK-8) solution (Bimake, Houston, TX, USA) was added to each well at the same time every day for 4 days. After a 3-h incubation, the absorbance was measured at 450 nm through a microplate reader (BioTek ELX800, Winooski, VT, USA).

Transfected cells were stained with the Ki-67 antibody (#ab15580, Abcam) for 30 min in the dark. Cells were washed twice with 40 ml phosphate-buffered saline/0.5% bovine serum albumin, resuspended in 5 ml phosphate-buffered saline/0.5% bovine serum albumin, and stained with the viability marker 7-AAD on ice for 10 min in the dark. The analysis was performed using BD FACS Diva. Sorting was performed by using a MoFlo high-speed cell sorter (Beckman Coulter).

Ethynyl deoxyuridine (Edu) incorporation was used to assess cell proliferation with EdU Kit (RiboBio) according to the manufacturer’s instructions. The cells were quantified with a fluorescence microscope (Olympus, Tokyo, Japan) and Image-Pro Plus 6.0 software.

For colony formation assays, cells (3 × 10^3^/well) were seeded in 12-well culture plates with three wells in each group. The cells were resuspended in 0.33% agar and nutrition was performed twice a week by adding two drops of MDS cell cultures to the medium. After incubation for 14 days at 37 °C, images of the 12-well plate were taken under a microscope then scanned, and counted the colony numbers with ImageJ.

### Cell cycle and apoptosis assay

For the cell cycle analysis, cells were fixed with 70% cold ethanol and stained with 0.1 mg/ml PI (Beyotime Biotechnology, Shanghai, China). Cell cycle distribution was analyzed by FACSCanto II flow cytometer (BD Biosciences San Diego, CA, USA). Apoptosis assay was performed with annexin V-FITC/PI Apoptosis Detection Kit (Vazyme Biotech) according to the manufacturer’s instructions. FlowJo software (vX.0.7, Ashland, OR) was used to analyzed data

### FISH and subcellular fractionation of BC200

To determine the subcellular location of BC200, locked nucleic acid-RNA fluorescence in situ hybridization (LNA-FISH) was performed with a FISH kit (RiboBio) according to the manufacturer’s protocol. LNA fluorescein-labeled probes against 18 S rRNA, U6 snoRNA, and BC200 were designed and synthesized by Ribo. Fluorescence signals were scanned using a Leica SP5 II scanning confocal microscope (Leica, Bannockburn, USA). A nucleus and cytoplasm segmentation PARIS™kit (Ambion, TX, USA) was used to segment the nucleus and cytoplasm of cells following the manufacturer’s instructions.

### Construction of the lncRNA-miRNA-mRNA regulatory network

The independent cohorts of primary MDS data of GSE114869 were downloaded from the Gene Expression Omnibus (GEO) database [13]. The relationship between differentially expressed lncRNAs (DElncRNAs) and DEmiRNAs was explored by the starBase v3.0 database based on CLIP-seq data research and the miRcode database based on the GENCODE database [22]. TargetScan, miRcode, and MiRanda were further used to determine the interactions between DEmiRNAs and DEmRNAs. Finally, according to the interactions between lncRNAs, miRNAs, and mRNAs, a ceRNA regulatory network was constructed and visualized by Cytoscape 3.6.1.

### Luciferase reporter assay

To evaluate promoter activity, BC200 promoter was cloned into GV238 vector. Then, HEK293T cells was cotransfected with GV238-BC200 and MYB siRNAs or control siRNA with Lipofectamine 3000 (Invitrogen). For 3'-UTR luciferase reporter assays, luciferase reporters were synthesized by cloning wild-type (wt) or mutant (mt) BC200 3'-UTR sequence containing the miR-150-5p binding site into GV272 vector. Cells were cotransfected with miR-150-5p mimics or control vector and GV272-BC200 or GV272-BC200-mt (miR-150-5p) using Lipofectamine 3000. Likewise, GV272-MYB or GV272-MYB-mt (miR-150-5p) was cotransfected with miR-150-5p mimics or control vector into HEK293T cells. After 48 h, firefly and Renilla luciferase activities were examed using the Dual-Luciferase Reporter Assay System (Promega, WI, USA) according to the manufacturer’s instructions. Luciferase activities were normalized to Renilla luciferase.

### ChIP assay

Pierce™ Magnetic chromatin immunoprecipitation (ChIP) Kit (Thermo Fisher Scientific) was used to perform ChIP assays. Briefly, MDS-L cells were crosslinked with 1% formaldehyde. Chromatin was isolated and immunoprecipitated with an MYB antibody (#05-175, Sigma-Aldrich, St Louis, MO, USA). Immunoprecipitated DNA was washed and eluted according to the manufacturer’s instructions. qRT-PCR was conducted to analyze the eluted DNA. The sequences of the primers used in ChIP assays are listed in Supplementary Table S3.

### RNA immunoprecipitation (RIP) assay

RIP was performed with a Magna RIP RNA-Binding Protein Immunoprecipitation Kit (Millipore, Billerica, MA, USA). In brief, MDS-L cells were collected and lysed in RIP buffer. Next, 100 µL cells containing magnetic beads were conjugated with human anti-Ago2 antibody (Abcam, Cambridge, MA, USA) or negative control (normal mouse IgG, Millipore). Finally, the immunoprecipitated RNA was detected by qRT-PCR to measure BC200 and miR-150-5p levels in the precipitates.

### RNA pull-down assay

A DNA fragment containing the full-length BC200 sequence or a negative control sequence was PCR amplified using T7 RNA polymerase (Roche, Basel, Switzerland). The resulting plasmid DNA was linearized using the restriction enzyme XhoI. Biotin-labeled RNA was reverse transcribed using Biotin RNA Labeling Mix (Roche) and T7 RNA polymerase (Takara Biomedical Technology). The products were treated with RNase-free DNase I (Roche) and purified with an RNeasy Mini Kit (Qiagen, MD, USA), with the resulting RNA used for real-time PCR assays. Moreover, the products were treated with Ago2 antibody (#03-110, Sigma-Aldrich) to detect cell lysates utilizing the sample pulled down by biotinylated BC200 and random probe.

### Western blot analysis

MDS cells were lysed with RIPA buffer (Vazyme Biotech) in the presence of a protease inhibitor cocktail (Vazyme Biotech, Nanjing). 50 μg of protein extracts were boiled and subject to 10% SDS-PAGE gel and then transferred to nitrocellulose membranes. The membranes were blocked by 5% non-fat dry milk (Bio-Rad, CA, USA) and incubated with mouse anti-human MYB (Sigma-Aldrich) and mouse anti-human GAPDH primary antibodies (#sc-32233, Santa Cruz Biotechnology, Dallas, TX, USA) (the dilutions of primary antibodies is 1:1000) at 4 °C overnight. The HRP-conjugated secondary antibodies were used at 1:3000 dilution for 1.5 h at room temperature. Signals were detected by ECL HRP substrate (Millipore).

### Tumorigenicity assays in vivo

All animal experiments were approved by the Animal Care and Use Committee of the Third Xiangya Hospital of Central South University (Changsha, China). All NOD-Prkdcem26Cd52Il2rgem26Cd22/NjuCrl (NCG) female mice, 4–6 weeks, were purchased from the Shanghai Lab Animal Research Center (Shanghai, China) and randomly divided into three groups (sample size: four mice per group). And investigators were blinded to the randomization. The right upper backs of NCG mice were injected subcutaneously with 5 × 10^6^ cells stably transfected with sh-BC200 mixed with 0.2 ml RPMI-1640 medium. The tumor volume for each mouse was measured every 2 days. On 26th day, the tumor-bearing mice were sacrificed, and the size of the tumors was measured and stored.

### Intravenous MDS model of NCG mice

The female NCG mice, 4–6 weeks, were randomly divided into three groups (sample size: three mice per group) and investigators were blinded to the randomization. SKM-1-vector (transfected with empty vector) and SKM-1 sh-BC200-1/2 (transfected with shRNA targeting BC200) cells (6 × 10^6^ cells per animal) were separately intravenously injected into the tail vein of NCG mice. After 1 month, the mice were sacrificed, and BM, peripheral blood, and tissue samples were collected.

### Fluorescence-activated cell sorting (FACS)

To analyze BM samples of intravenous MDS mice by flow cytometry, the BM cells were stained with the following anti-human antibodies: CD34-APC (#343608, BD Pharmingen), CD45-APC-Cy (#304014, BD Pharmingen), CD71-PE (#334106, BD Pharmingen), glycophorin A (GPA)–BV421 (#562938, Biolegend), CD36-BV605 (#563518, Biolegend), CD38-FITC (#356610, Biolegend), anti–IL-3R (CD123)-PE-Cy7 (#25-1239-42, Invitrogen) for 30 min in the dark. Cells were washed twice with 40 ml phosphate-buffered saline/0.5% bovine serum albumin, resuspended in 5 ml phosphate-buffered saline/0.5% bovine serum albumin, and stained with the viability marker 7-AAD on ice for 10 min in the dark. The analysis was performed using BD FACS Diva. Sorting was performed by using a MoFlo high-speed cell sorter (Beckman Coulter).

### Peripheral blood cells count

After inoculating the NCG mice with SKM-1 cells via the tail vein for 30 days, blood samples were collected into ethylenediaminetetraacetic acid-coated tubes and full blood counts were determined on an Advia 120 automated hematology analyzer.

### Immunohistochemical (IHC)

The paraffin-embedded sections were from tumors and tissues of mice used for evaluating the expression of MYB and Ki-67 protein. Immunohistochemical staining used a streptavidin-peroxidase method according to the manufacturer’s introduction.

### Bioinformatics analysis

Two primary MDS datasets cohorts GSE114869 and GSE99095 were downloaded from the GEO database. GSE114869 has 300 primary MDS samples and 20 normal MDS samples; GSE99095 comes from the single-cell RNA sequencing for five primary MDS samples and four normal MDS samples. GSE114869 and GSE99095 gene expression profile data were analyzed by means of Significant Analysis of Microarray (SAM) software.

### Statistical analysis

Student’s *t*-test was used to determine the significance of the differences between the control and the experimental groups using the SPSS 22.0 statistical software and GraphPad Prism 8.0 (GraphPad software). Values were shown as mean ± SD for three independent experiments. *P* < 0.05 was considered to indicate statistical significance. The correlation analysis was performed using Pearson’s correlation test. *P* < 0.05 was considered to indicate statistical significance.

## Results

### Knockdown of BC200 inhibits the proliferation of MDS cells

**Fig. 1:**
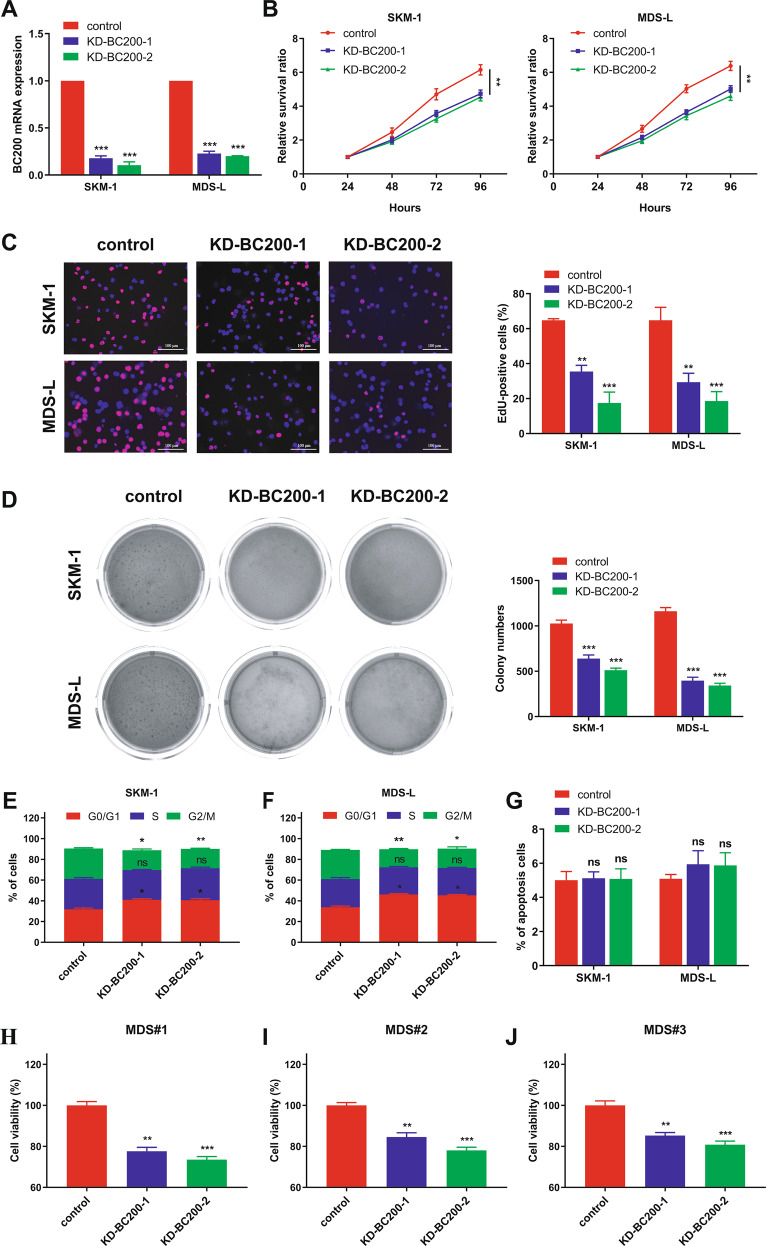
Knockdown of BC200 inhibits the proliferation of MDS cells. **A** In SKM-1 and MDS-L cells, the transcriptional level of BC200 was downregulated by two shRNAs. **B–D** MDS cells proliferation was detected by CCK-8, EdU incorporation (Scale bar, 100 μm) and colony formation assays. It revealed that BC200 knockdown significantly suppressed SKM-1 and MDS-L cell proliferation. **E**, **F** Knockdown of BC200 led to G0/G1 arrest in both SKM-1 and MDS-L cells. **G** Apoptosis assay showed that the percentage of apoptotic MDS cells was not affected by BC200 knockdown. **H–J** Human primary BMMCs were treated with BC200 siRNAs for 72 h, and cell proliferation was analyzed by the CCK-8 assay. *p < 0.05, **p < 0.01, ***p < 0.001, ns, not significant.

To explore the role of BC200 in the proliferation of MDS cells, we selected two shRNA with the most effective interference effect and confirmed the knockdown effect in SKM-1 and MDS-L cells (Fig. 1A). Cell proliferation was analyzed by CCK-8, Edu incorporation, colony formation assays, and FACS analysis. Knockdown of BC200 significantly suppressed MDS cell viability, which was detected by CCK-8 and Ki-67 (Fig. 1B and Fig. S1A). Edu incorporation and colony formation assays also indicated that knockdown of BC200 also inhibited the malignant proliferation ability of SKM-1 and MDS-L cells compared to the paired negative control shRNA (Fig. 1C, D). Notably, we explored whether knockdown of BC200 suppressed the proliferation of MDS-L and SKM-1 cells by inducing cell cycle arrest at G0/G1 phase rather than cell apoptosis (Fig. 1E–G and Fig. S1B, C). The results indicated that knockdown of BC200 by two BC200 siRNAs inhibited the proliferation of primary bone marrow mononuclear cells (BMMCs) obtained from MDS patients and cultured in vitro (Fig. 1H–J).

### BC200 acts as a molecular sponge for miR-150-5p in MDS cells

**Fig. 2:**
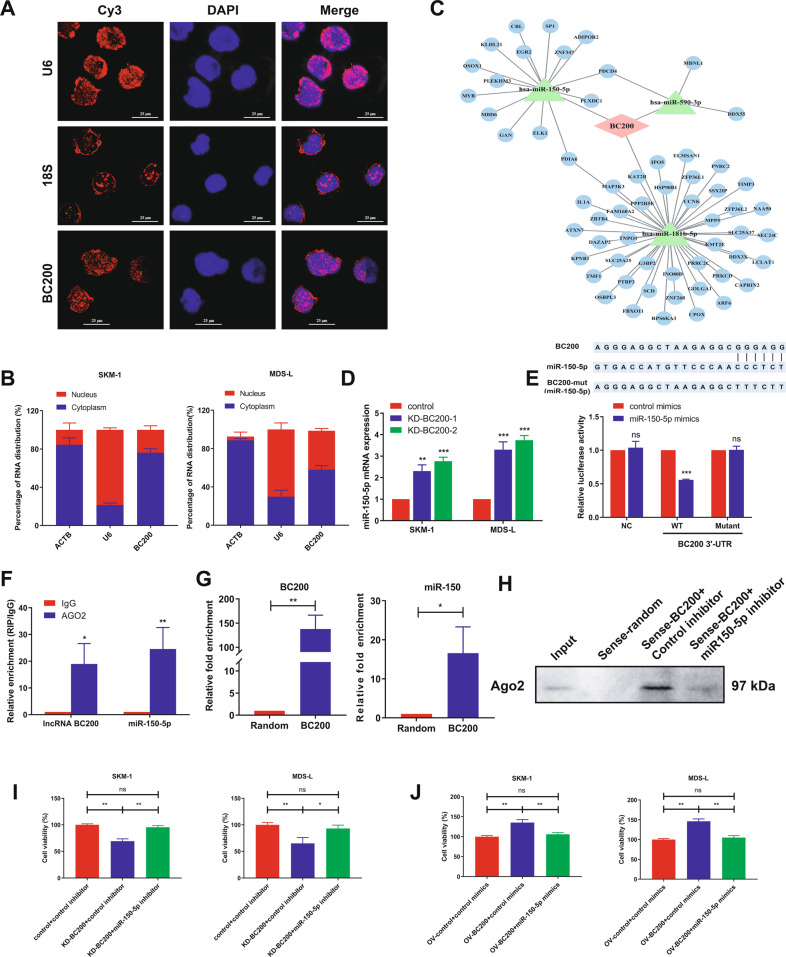
BC200 acts as a molecular sponge for miR-150-5p in MDS cells. **A** RNA FISH assays for BC200 in SKM-1 cells. Stain the nucleus with DAPI. Scale bar, 25 μm. **B** BC200, U6, and ACTB expression in the RNA extracted from the cytoplasm and nucleus of MDS cells by qRT-PCR. **C** The ceRNA regulatory network of BC200 and three miRNAs containing the binding site of BC200. **D** Transcriptional level of miR-150-5p was detected in sh-BC200 MDS cells. **E** Upper: complementary sequence between miR-150-5p and BC200-wt. The putative binding sites of miR-150-5p in BC200-mt. Lower: Luciferase activity was measured in HEK293T cells cotransfected with miR-150-5p mimics and wt or mt BC200 vector. **F** RIP assays of the enrichment of Ago2 on BC200 and miR-150-5p relative to IgG in MDS-L cells. The relative expression levels of BC200 and miR-150-5p were detected by qRT-PCR. **G** Detection of BC200 or miR-150-5p by using qRT-PCR in the sample pulled down by biotinylated BC200 and Random probe. Input was used for normalization. **H** The correlation among BC200, miR-150-5p, and Ago2 was determined by using Ago2 antibody to detect cell lysates utilizing the sample pulled down by biotinylated BC200 and Random probe. **I** CCK-8 assay rescue experiment showed that cell proliferation reduced by si-BC200 could be increased by miR-150-5p inhibitor in MDS cells. **J** CCK-8 assay rescue experiment showed that cell proliferation stimulated by overexpression of BC200 could be suppressed by miR-150-5p mimics in MDS cells. *p < 0.05, **p < 0.01, ***p < 0.001, ns, not significant.

As indicated in previous reports, the location of lncRNAs frequently determines their functions [23]. In this study, to investigate the mechanisms by which BC200 functions as a carcinogen in MDS, we first determined its location in SKM-1 cells. As the FISH and subcellular fractionation data show, BC200 was mainly located in the cytoplasm of SKM-1 cells, indicating that BC200 plays a regulatory role in MDS cells through the ceRNA network (Fig. 2A, B). We further analyzed the DElncRNAs in the RNA sequencing data of a GEO dataset (GSE114869) and generated a ceRNA network diagram (Fig. S2A). We separately analyzed the ceRNA regulatory network of BC200 and predicted three miRNAs (miR-150-5p, miR-181b, and miR-590-3p) containing binding site of BC200 (Fig. 2C). Interestingly, knockdown of BC200 significantly increased miR-150-5p expression in MDS cells rather than miR-181b and miR-590-3p (Fig. 2D and Fig. S2B, C), indicating that miR-150-5p may be the functional downstream molecule of BC200. Next, a luciferase reporter assay in HEK293T cells showed that miR-150-5p overexpression reduced the luciferase activity of GV272-BC200 but did not influence that of GV272-BC200-mt (Fig. 2E). To verify whether BC200 and miR-150-5p were associated with the RNA-induced silencing complex (RISC), RIP assays were performed utilizing an antibody against Ago2 (the core component of the RISC). It revealed that both BC200 and miR-150-5p were drastically enriched in Ago2 pellets compared with IgG immunoprecipitates in MDS-L cells (Fig. 2F), suggesting that BC200 was physically present in the Ago2-based miRNA-induced repression complex and was associated with miR-150-5p. Subsequently, the biotin-labeled pull-down results showed a significant amount of BC200 and miR-150-5p in the BC200 pull-down pellet compared with that observed in the control group as measured by qRT-PCR (Fig. 2G, H). These data indicated that BC200 could negatively regulate miR-150-5p expression in MDS cells.

To ascertain whether miR-150-5p could affect MDS cell proliferation, we transfected miR-150-5p mimics and miR-150-5p inhibitors in MDS cells. The CCK-8 results showed that miR-150-5p mimics suppressed MDS cell proliferation after transfection compared to the control mimics (Fig. S2D). In contrast, the miR-150-5p inhibitor promoted MDS cell proliferation after transfection (Fig. S2E). Furthermore, overexpression of miR-150-5p significantly suppressed MDS cell viability detected by Ki-67 (Fig. S2F). Notably, miR-150-5p mimics suppressed the proliferation of MDS-L and SKM-1 cells by inducing cell cycle arrest at S phase and promoting cell apoptosis (Fig. S2G, H).

Moreover, inhibition of miR-150-5p rescued the suppressive effect of BC200 knockdown by siRNA on MDS cells (Fig. 2I), while overexpression of miR-150-5p attenuated the increased proliferation abilities of MDS cells overexpressing BC200 (Fig. 2J). Thus, these results strongly suggested that the oncogenic effect of BC200 on MDS was partially mediated by miR-150-5p.

### MYB is a direct target of miR-150-5p in MDS cells

**Fig. 3:**
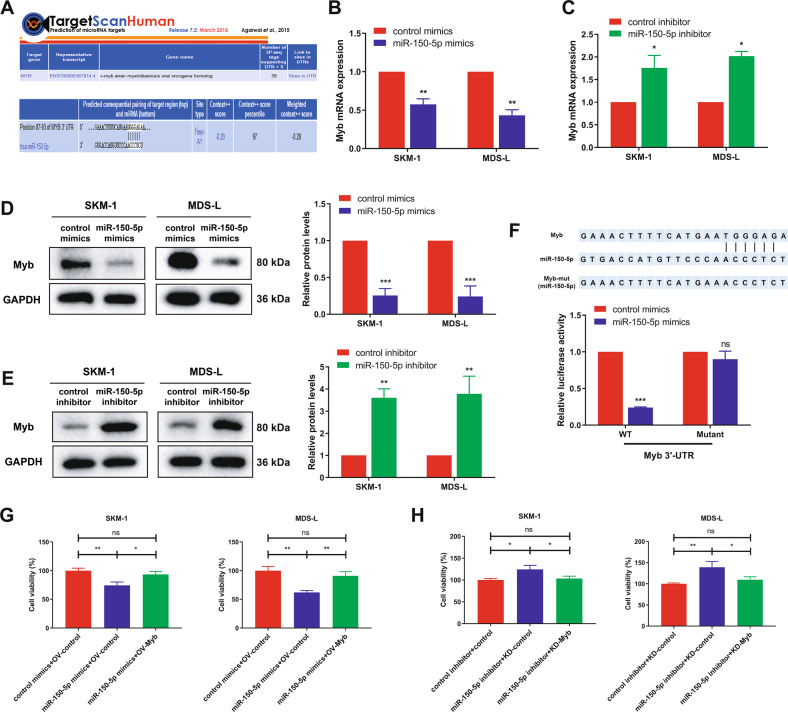
MYB is a direct target of miR-150-5p in MDS cells. **A** MYB was the top candidate target containing the complementary site for the seed region of miR-150-5p in the TargetScan database. **B**, **C** SKM-1 and MDS-L cells were transfected with miR-150-5p mimics or inhibitors for 48 h, and MYB mRNA levels were detected by qRT-PCR. **D**, **E** MYB protein expression in both SKM-1 and MDS-L cells transfected with miR-150-5p mimics or inhibitor for 48 h was detected by western blotting. **F** Upper: putative MYB binding sites on the promoter region of miR-150-5p and their corresponding mutant binding sites. Lower: luciferase activity in HEK293T cells cotransfected with miR-150-5p or control mimics and luciferase reporters containing the wt or mt MYB 3'-UTR. **G** CCK-8 assay detected the effect of MYB overexpression and miR-150-5p mimics on SKM-1 and MDS-L cell proliferation activity after 72 h. **H** CCK-8 assay detected the effect of MYB knockdown and miR-150-5p inhibitor on SKM-1 and MDS-L cell proliferation activity after 72 h. *p < 0.05, **p < 0.01, ***p < 0.001, ns, not significant.

To explore into the molecular mechanisms for miR-150-5p suppressing MDS cell proliferation, starBase v3.0 and TargetScan bioinformatic analyses were conducted to forecast the putative targets of miR-150-5p. Among the targets, MYB, which ranked number one, had a conserved miR-150 binding site and was identified as a potential target of miR-150-5p (Fig. 3A). Previously, MYB was reported as a direct target of miR-150-5p in hepatocellular carcinoma [24, 25]. Our results showed that overexpression of miR-150-5p markedly downregulated MYB at the mRNA and protein levels, while inhibition of miR-150-5p upregulated the expression of MYB in SKM-1 and MDS-L cells (Fig. 3B–E). To confirm whether MYB is a target gene for miR-150-5p, a luciferase reporter assay was performed. In HEK293T cells, overexpression of miR-150-5p reduced the luciferase activity of GV272-MYB but did not influence that of GV272-MYB-mt (Fig. 3F). Moreover, we examined the function of MYB in MDS cell proliferation. As shown in Supplementary Fig. S3A–D, silencing MYB in MDS cells by using two siRNAs significantly suppressed MDS cell proliferation.

To further verify that MYB is a functional target of miR-150-5p and is responsible for promoting cell proliferation, miR-150-5p mimics/inhibitor and MYB plasmid/siRNA were introduced alone or simultaneously into MDS cells. Overexpression of MYB restored the inhibitory effect of miR-150-5p mimics on MDS cell proliferation (Fig. 3G). Conversely, MYB knockdown attenuated the miR-150-5p inhibitor-induced proliferation of MDS cells (Fig. 3H). Hence, these findings demonstrated that miR-150-5p regulated the proliferation of MDS cells by targeting MYB expression.

### BC200 promotes the malignant proliferation of MDS cells via the miR-150-5p/MYB axis

**Fig. 4:**
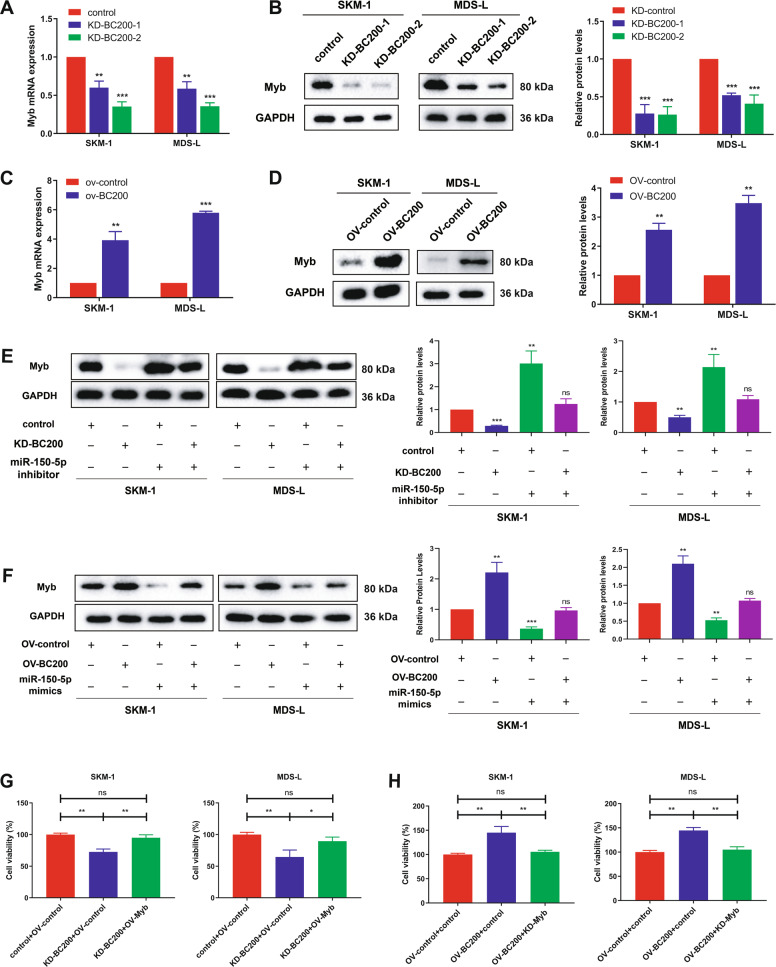
BC200 promotes the proliferation of MDS cells via the miR-150-5p/MYB axis. **A** The mRNA levels of MYB were measured in sh-BC200 MDS cells. **B** The protein expression levels of MYB were measured in MDS cells transfected with two different BC200 shRNAs. **C**, **D** qRT-PCR and western blotting were used to measure the MYB mRNA and protein levels in SKM-1 and MDS-L cells transfected with BC200 plasmid (ov-BC200) for 48 h. **E**, **F** Western blot analysis of MYB in MDS cells cotransfected with miR-150-5p mimics and BC200 siRNA or miR-150-5p inhibitors and BC200 plasmid for 48 h. **G** CCK-8 assay indicated the effect of BC200 overexpression and MYB suppression on SKM-1 and MDS-L cell proliferation activity after 72 h. **H** CCK-8 assay indicated the effect of BC200 knockdown and MYB upregulation on SKM-1 and MDS-L cell proliferation activity after 72 h. *p < 0.05, **p < 0.01, ***p < 0.001, ns, not significant.

It was further confirmed that BC200 promoted the proliferation of MDS cells via the miR-150-5p/MYB axis, and BC200 negatively regulated miR-150-5p. We found that knockdown of BC200 reduced both MYB mRNA and protein levels (Fig. 4A, B), while overexpression of BC200 significantly increased the mRNA and protein levels of MYB (Fig. 4C, D). Furthermore, the upregulation of MYB induced by BC200 overexpression was abrogated by miR-150-5p mimics, and similarly, the reduction in MYB levels induced by knockdown of BC200 by siRNA was reversed by the miR-150-5p inhibitor in both SKM-1 and MDS-L cells (Fig. 4E, F). On the other hand, overexpression of MYB completely reversed the cell proliferation-inhibiting effects of BC200 siRNA, while the increase in MDS cell proliferation by BC200 overexpression was abolished by knockdown of MYB (Fig. 4G, H). Thus, our findings indicated that BC200 promoted MDS malignant proliferation through the miR-150-5p/MYB axis.

### MYB transcriptionally regulates BC200 expression in MDS cells

**Fig. 5:**
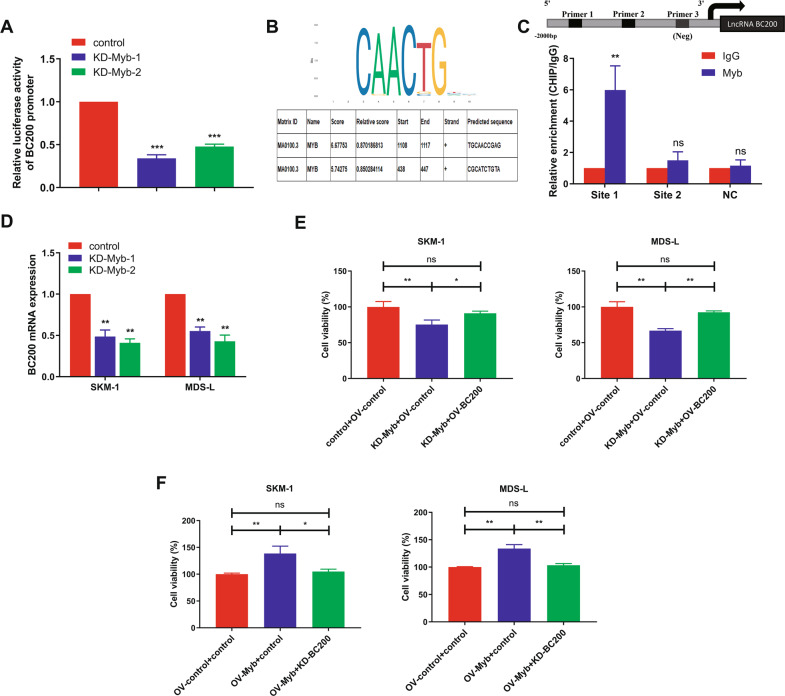
MYB transcriptionally regulates BC200 expression in MDS cells. **A** HEK293T cells were cotransfected with GV238-BC200 and MYB siRNAs for 48 h. **B** The MYB binding sitein BC200 predicted by JASPAR matrix models. **C** Upper: putative MYB binding sites BC200 promoter region and design of the indicated primers. Lower: ChIP assays of the enrichment of MYB on the BC200 promoter relative to control IgG in MDS-L cells. **D** The expression level of BC200 was detected in MDS cells transfected with two different MYB siRNAs. **E**, **F** SKM-1 and MDS-L cells were treated with the indicated siRNAs or plasmids for 72 h. The cell proliferative was measured by CCK-8 assay. *p < 0.05, **p < 0.01, ***p < 0.001, ns, not significant.

To understand the potential mechanism of the abnormally high expression of BC200 in MDS, we used the ChIP-seq data from the public UCSC Genome Browser (http://genome.ucsc.edu/index.html) to identify BC200 potential transcription factors. Interestingly, the transcription factor MYB had been identified as potentially binding to the upstream regulatory region of the BC200 gene (Fig. S4). To verify MYB could regulate BC200 in MDS, we proved that knockdown of MYB significantly reduced BC200 expression (Fig. 5A). To further determine the transcriptional activity of MYB on the BC200 gene promoter, GV238-BC200 and MYB siRNAs were cotransfected into cells. As shown in Fig. 5A, knockdown of MYB markedly reduced BC200 promoter activity. Furthermore, it has been predicted that the transcription factor MYB has two binding sites on the BC200 promoter, “TGCAACCGAG” (Site 1) and “CGCATCTGTA” (Site 2) using bioinformatics analyses (JASPAR, ifti.org) (Fig. 5B). ChIP assays demonstrated the significant enrichment of MYB on binding Site 1 of BC200 promoter (Fig. 5C). In further studies of the effect of MYB on BC200 transcription in MDS, we found that knockdown of MYB significantly reduced BC200 expression in SKM-1 and MDS-L cells (Fig. 5D). More importantly, rescue assays were conducted to determine whether MYB promotes malignant proliferation through BC200 in MDS cells. We found that MYB suppression decreased cell proliferation, whereas BC200 overexpression markedly rescued this phenotype (Fig. 5E). In contrast, MYB overexpression increased cell proliferation, whereas BC200 suppression markedly rescued this phenotype (Fig. 5F). Taken together, these results illustrated that the BC200/miR-150-5p/MYB positive feedback loop promotes malignant proliferation in MDS cells.

### The BC200/miR-150-5p/MYB loop promotes tumor growth in vivo

**Fig. 6:**
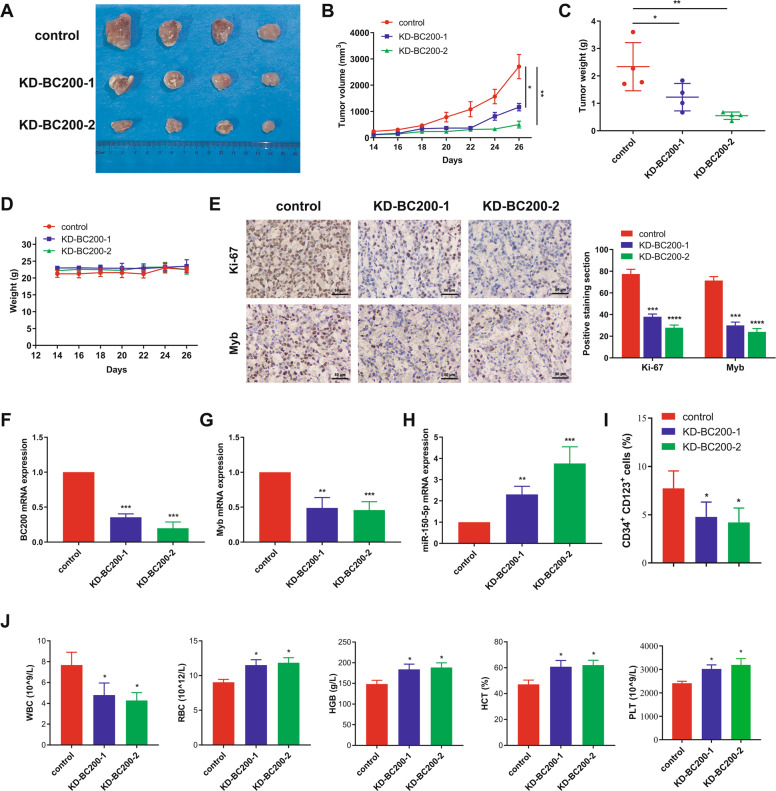
Knockdown of BC200 inhibits MDS growth in vivo. **A** SKM-1 cells treated with different BC200 shRNAs (*n* = 4) or control shRNA (*n* = 4) were inoculated subcutaneously into NCG mice. Tumor growth curves showed that sh-BC200 group led to tumor growth restriction in mice. Scale bar: 1 cm. **B** Tumor growth curves showed that sh-BC200 group suppressed tumor growth compared with sh-control group. **C** The subcutaneous tumors were harvested and weighed on the 26th day after implantation. Data are shown as mean ± SEM. **D** The NCG mice grew tumors on the fourteenth day after implantation and began to be weighed. **E** Ki-67 immunostaining of xenograft tissues collected from the BC200-knockdown group and control group. Scale bar: 50 μm. **F–H** qRT-PCR was performed on xenograft tissues and was subjected to measure BC200, MYB, and miR-150-5p expression. **I** Plot of CD123 vs CD34 expression of BM cells of MDS or normal mice being analyzed. **J** White blood cells (WBCs), red blood cells (RBCs), hemoglobin (HGB), hematocrit (HCT), and platelets (PLTs) counts of MDS or normal mice. *p < 0.05, **p < 0.01, ***p < 0.001, ****p < 0.0001.

We next explored the pro-proliferative role of the BC200/miR-150-5p/MYB loop in vivo. First, subcutaneous xenografts were established in the flanks of NCG mice using sh-BC200-1/2 cells and sh-control cells. The volume (Fig. 6A, B) and weight (Fig. 6C) of BC200-silenced tumors were markedly smaller than those of tumors formed by control cells, while the mouse body weights were not significantly different between the BC200-silenced groups and the control group (Fig. 6D), indicating that sh-BC200 had no obvious side effects on the mice. In addition, the immunohistochemical staining results showed that both Ki-67 and MYB were expressed at low levels in BC200-silenced tumor tissues (Fig. 6E). qRT-PCR data showed that the mRNA levels of BC200 and MYB were downregulated, while miR-150-5p was upregulated in BC200-silenced tumor tissues compared with control tissues (Fig. 6F–H). Collectively, these results demonstrated that the BC200/miR-150-5p/MYB loop promoted the tumor growth of MDS cells in vivo.

Subsequently, we established the intravenous MDS mouse model by injecting sh-BC200-1/2 cells and sh-control cells. BC200 knockdown markedly reduced CD123^+^CD34^+^ cells, which was related to the malignant clonal cells with aberrant differentiation, excessive proliferation, and decreased apoptosis in MDS (Fig. 6I and Fig. S5A). CD36^+^ and CD38^+^ BM cells were not significantly different between the BC200-silenced groups and the control group, suggesting that there was no significant change in granulocyte-monocyte and lymphoid lineage in BC200 knockdown BM cells (Fig. S5B–D). Moreover, Flow cytometry showed decreased human CD71^−^GPA^−^, while increased CD71^−^GPA^+^ in BM cells of intravenous BC200-silenced groups, suggesting promoted differentiation from the undifferentiated progenitor cells to mature erythrocytes (Fig. S5E). Correspondingly, peripheral blood cells analysis further revealed that knockdown BC200 significantly increased red blood cells (RBCs), hemoglobin (HGB), hematocrit (HCT), and platelets (PLTs) and decreased white blood cells (WBCs) (Fig. 6J). In addition, the immunohistochemical staining results showed that the expression level of Ki-67 in the spleen of BC200-silencing group decreased slightly compared with the control group (Fig. S5F).

### Expression and clinical significance of the BC200/miR-150-5p/MYB loop in MDS samples

**Fig. 7:**
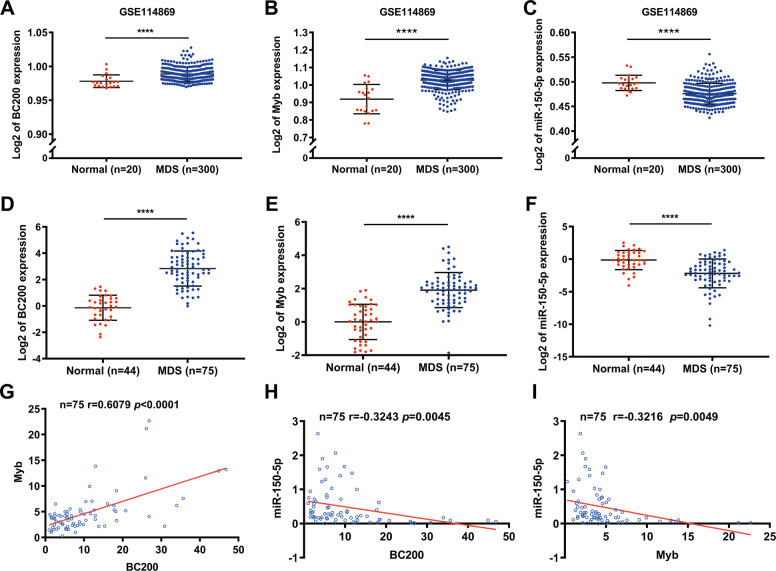
Clinical significance of the BC200/miR-150-5p/MYB loop based on bioinformatics analysis. **A–C** Analysis of a GEO dataset (GSE114869) indicated that BC200 and MYB expression was markedly higher in MDS samples than in normal samples. Conversely, miR-150-5p was significantly lower in MDS samples. **D–F** The expression of BC200, miR-150-5p mRNA, and MYB was analyzed by qRT-PCR in 79 MDS samples and 44 normal samples. **G–I** Correlation analysis of BC200 and miR-150-5p, MYB and miR-150-5p, and BC200 and MYB expression levels in 75 MDS samples. ****p < 0.0001.

To determine the potential clinical significance of the BC200/miR-150-5p/MYB loop in MDS, we systematically analyzed the expression profiles of these genes in MDS samples versus normal samples using two published GEO datasets (GSE114869 and GSE99095) [26]. Analysis of the GEO data repository showed that the expression levels of both BC200 and MYB were dramatically higher, while miR-150-5p was significantly lower in MDS samples than in normal samples (Fig. 7A–C and Fig. S6A). Furthermore, the expression of the BC200/miR-150-5p/MYB loop was detected by qRT-PCR in our collected normal and MDS patient BMMC samples, and the results showed that their expression levels were consistent with the results of the analysis of MDS patients from the TCGA dataset (Fig. 7D–F), and the expression levels of these molecules were well correlated (Fig. 7G–I). In addition, qRT-PCR revealed that the expression of BC200/miR-150-5p/MYB in human MDS cell lines compared with human normal BMMCs was similar to that in the GEO datasets (Fig. S6B–D). Taken together, these results indicated that the dysregulated expression of BC200/miR-150-5p/MYB was a frequent event in MDS and that this positive feedback loop potentially promoted the pathogenesis and development of MDS.

## Discussion

Several studies have shown that dysregulated lncRNAs play essential roles in the occurrence and development of MDS [14, 27, 28]. In addition to the well-characterized lncRNAs, it is worth exploring potential essential lncRNAs that control the pathogenesis and development of MDS. Thus, we analyzed publicly available databases of DElncRNAs in MDS. Interestingly, we found that carcinogenic BC200 was highly expressed in BMMCs of MDS patients compared with those of normal individuals. Numerous studies have also indicated that BC200 functions as an oncogenic lncRNA, and it has been shown to play important roles in the growth, migration, and invasion of cancer cells. BC200 has become an emerging novel diagnostic marker and therapeutic target in human cancers [16, 29, 30]. Therefore, the abnormal expression and biological function of BC200 in MDS are worth investigating.

Normal cell cycle progression is essential for cell proliferation and differentiation. Disruption or dysregulation of the cell cycle leads to unlimited cell proliferation and inhibition of differentiation and apoptosis, which is closely related to tumorigenesis. Therefore, we further explored the potential function and mechanism of BC200 in MDS tumorigenesis. Loss-of-function assays showed that knockdown of BC200 suppressed proliferation, colony formation, and cell cycle progression but could not induce apoptosis of MDS cells. In vivo experiments demonstrated that BC200 inhibition suppressed the growth and invasiveness of MDS cells in mice. These results indicated that BC200 plays an important role in the pathogenesis and development of MDS. Moreover, we identified MYB as a novel downstream gene of BC200. Our findings revealed a complex circuitry underlying the concomitant upregulation of BC200 and MYB in MDS cells. Therefore, these findings are of great significance to explore the pathogenesis and treatment of MDS.

In recent years, a new topic of interest in the RNA field has become the ceRNA theory, which proposes a regulatory network between different RNAs, including lncRNAs and miRNAs [31]. As ceRNAs, lncRNAs sponge miRNAs through sequence complementarity and subsequently affect the functional roles of miRNAs [32]. A recent study found that BC200 could regulate CUEDC2 expression and the PTEN/AKT/p21 pathway by functioning as a ceRNA for sponging miR-619-5p to inhibit glioma progression [33]. In this study, we found that BC200 was mainly located in the cytoplasm. We hypothesized that BC200 regulates MYB by acting as a ceRNA to sponge miRNAs. Bioinformatics analysis and luciferase reporter assays revealed that miR-150-5p is a target of BC200. Moreover, BC200 silencing markedly upregulated miR-150-5p expression in MDS cells, which confirmed our hypothesis. Previously, miR-150-5p was found to be downregulated in various cancers and has been identified as a tumor suppressor [34, 35]. However, Hussein K et al. reported that miR-150 was upregulated in MDS-del (5q), and inhibition of proliferation might contribute to myelodysplastic hematopoiesis via overexpressing miR-150 [36]. The precise functions and underlying molecular mechanisms of miR-150-5p in the context of MDS are still unclear. We demonstrated that overexpression of miR-150-5p in MDS-L and SKM-1 cells could inhibit cell proliferation and induce cell cycle arrest at the G1/G0 phase. Our results revealed that the interaction between BC200 and miR-150-5p plays an important role in MDS and has clinical significance for MDS tumorigenesis, and BC200 exerts its oncogenic effects partly by sponging miR-150-5p in MDS cells.

We further identified the target of miR-150-5p by using bioinformatics analysis and luciferase reporter assays and confirmed MYB as a direct target of miR-150-5p in MDS cells. MYB has been reported to be regulated by miR-150-5p in many disorders [37, 38]. As an essential hematopoietic regulatory transcription factor, MYB is crucial for the proliferation and differentiation of hematopoietic stem cells [39–41]. Dysregulation of MYB activity is often associated with various hematological disorders [42, 43]. Notably, in a zebrafish model, hyperactivity of MYB displayed MDS phenotypes similar to those in human patients [41].

In this study, we found that overexpression of BC200 in MDS sponged miR-150-5p and further resulted in an increase in MYB mRNA levels. Furthermore, we revealed that MYB was increased in MDS cells and that knockdown of MYB significantly inhibited the proliferation of MDS cells and diminished the proliferation-promoting effect of the miR-150-5p inhibitor in these cells. Therefore, we confirmed that MYB was the functional target of BC200/miR-150-5p in MDS.

Finally, we explored the molecular mechanism by which BC200 is upregulated in MDS. Previous studies have reported that the transcription factor MYB transcriptionally regulates its target genes to act as the critical driver in many biological processes, such as cell proliferation, metastasis, and differentiation [44, 45]. MYB upregulated miR-130a expression and activated the STAT3 and AKT pathways by downregulating NDRG2, which promotes proliferation and metastasis in salivary adenoid cystic carcinoma (SACC) [44]. Moreover, c-MYB could enhance invasion and metastasis through the wnt/β-catenin/axin2 pathway in breast cancer [46]. In acute myeloid leukemia (AML) with inv(3)/t(3;3), it was also found that targeting MYB or mutating its DNA-binding motif within the *GATA2* enhancer resulted in myeloid differentiation and cell death, suggesting that interference with transcription of MYB downstream gene *EVI1* provides a potential entry point for AML therapy [47]. In addition to a large number of protein-coding genes, many non-coding RNAs, including lncRNAs and miRNAs, are downstream targets of MYB. Lan T et al. reported that MYB enhanced lncRNA SNHG10 expression by regulating SNHG10 promoter activity, which affected SNHG10-driven HCC cell proliferation, invasion, and migration [24]. Herein, we showed that BC200 was a novel transcriptional target of MYB, and ChIP and dual-luciferase reporter assays demonstrated that MYB directly transcriptionally regulated BC200. Overexpression of MYB positively regulated BC200 abundance and further participated in the malignant growth of MDS cells. Importantly, MYB reversed the oncogenic effect of BC200 on MDS cells. Clinically, we demonstrated that the expression levels of both BC200 and MYB were dramatically higher, while miR-150-5p was significantly lower in MDS samples than in normal samples based on published GEO datasets and our collected patient samples, and the expression levels of these molecules were well correlated.

**Fig. 8:**
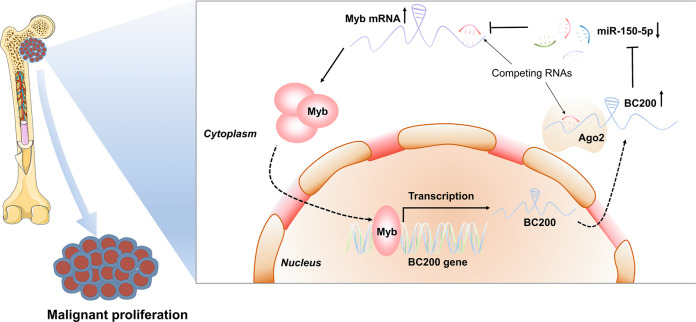
Schematic diagram of the BC200/miR-150-5p/MYB positive feedback loop in promoting the proliferation of MDS cells. BC200 acts as a miRNA sponge and positively regulates the expression of MYB through sponging miR-150-5p. Reciprocally, MYB can transcriptionally regulate the expression of BC200, and the overexpression of MYB enhanced the oncogenic effect of BC200/miR-150-5p. Therefore, BC200/miR-150-5p/MYB positive feedback loop is involved in promoting the malignant proliferation of MDS cells.

In conclusion, our study revealed that BC200 is a novel MYB-activated lncRNA. BC200 was highly expressed and played an oncogenic role in MDS cells and mouse models. Mechanistically, BC200 sponged miR-150-5p to attenuate its repressive effect on MYB and promoted the malignant proliferation of MDS cells. This study provided new insight into the function and molecular mechanism of the BC200/miR-150-5p/MYB positive feedback loop in MDS (Fig. 8), indicating that disrupting the BC200/miR-150-5p/MYB loop may be a novel therapeutic approach for MDS.

## Supplementary information

Supplemental Material

Reproducibility checklist

## Data Availability

The datasets used and/or analyzed during the current study are available from the corresponding author on reasonable request.
