# Supplementary material for: LncRNA BC200/miR-150-5p/MYB positive feedback loop promotes the malignant proliferation of myelodysplastic syndrome
Source: Cell Death Dis. 2022 Feb 8;13(2):126. doi: 10.1038/s41419-022-04578-2 (PMC8825806; doi:10.1038/s41419-022-04578-2)
Supplement: Supplementary file 1 — Supplemental Material [file 41419_2022_4578_MOESM1_ESM.docx]

**Supplementary Figures and Tables**

**Supplementary Figure S1**


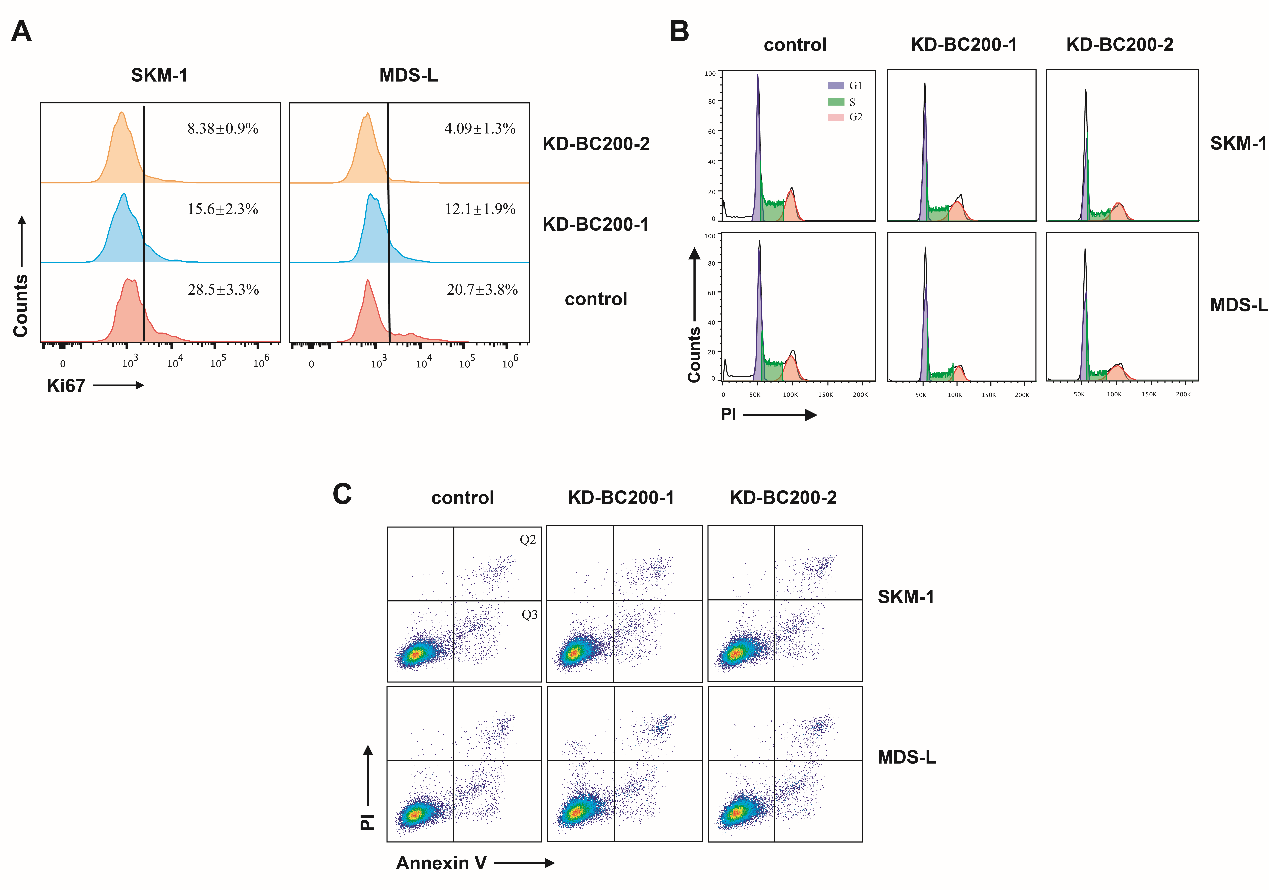


**Figure S1. BC200 promotes the proliferation of in MDS cells.**

**A** FACS was used to quantify percentage of Ki67 positive cells and assess the effect of BC200 knockdown on cell proliferation. **B** Cell cycle was applied to detect cell proliferation after the transfection of sh-BC200 in SKM-1 and MDS-L compared to sh-control. **C** Apoptosis assay showed that the percentage of apoptotic MDS cells was not affected by BC200 knockdown.

**Supplementary Figure S2**

**
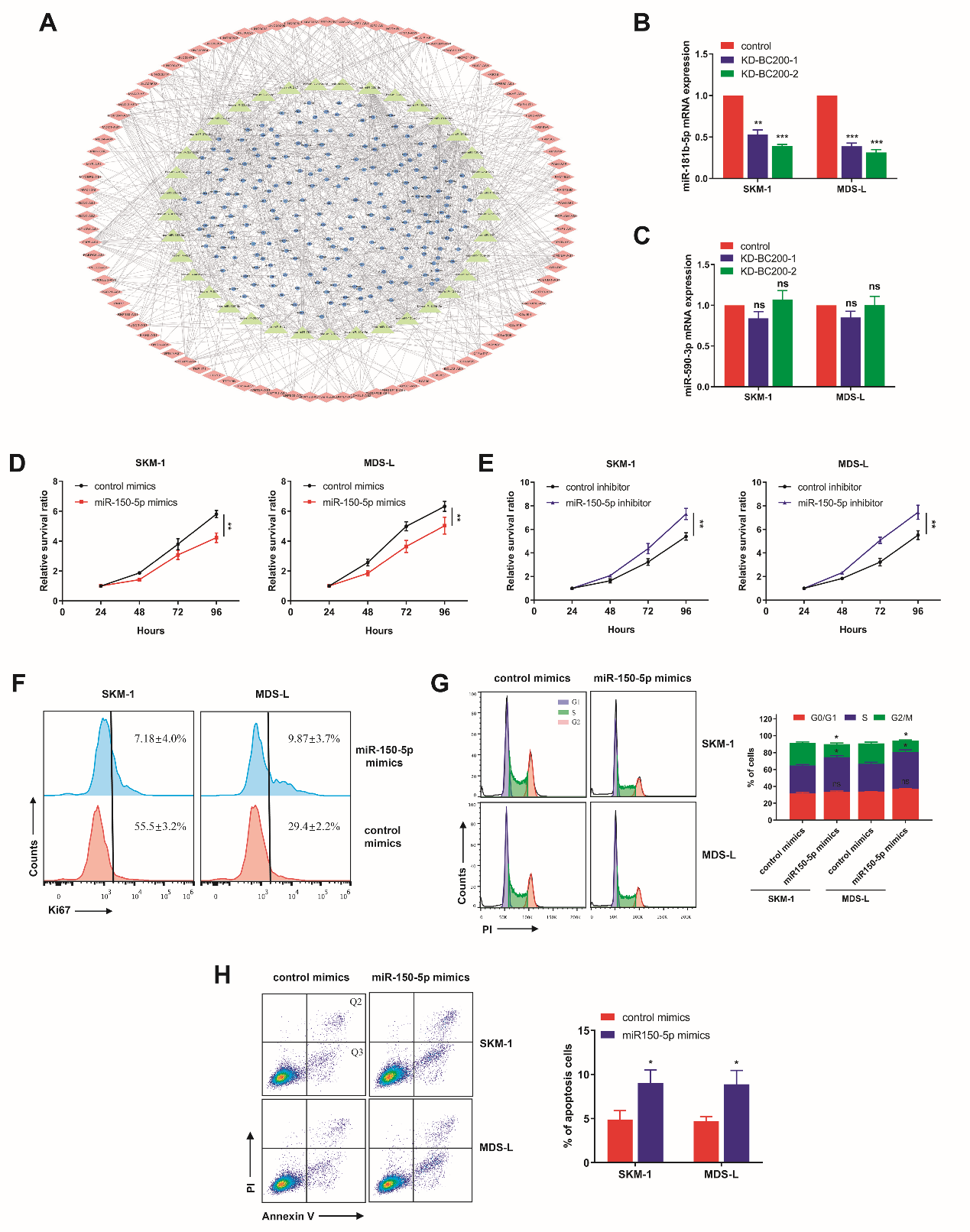
**

**Figure S2. A ceRNA network diagram of MDS and validating the ceRNA of BC200.**

**A** The relationship between differentially expressed lncRNAs (DElncRNAs) and miRNAs (DEmiRNAs) of GSE114869 were determined by the starBase v3.0 database, while the interactions between DEmiRNAs and DEmRNAs were predicted through TargetScan, miRcode and MiRanda. According to the predicted lncRNA, miRNA and mRNA interactions, a ceRNA regulatory network was constructed and visualized by Cytoscape 3.6.1. **B** and **C** SKM-1 and MDS-L cells transfected with two different BC200 shRNAs or control shRNA were subjected to qRT-PCR to measure miR-181b-5p and miR-590-3p expression. **D** and **E** CCK-8 assays showed that overexpression of miR-150-5p significantly inhibited the proliferation of MDS cells, while inhibition of miR-150-5p promoted the proliferation of MDS cells. **F** FACS was used to quantify percentage of Ki67 positive cells and assessed the effect of miR-150-5p overexpression on cell proliferation. **G** Overexpression of miR-150-5p led to S phase arrest in both SKM-1 and MDS-L cells. **H** The percentage of apoptotic MDS cells was increased after BC200 knockdown. **p* < 0.05, ***p* < 0.01, ****p* < 0.001, ns, not significant.

**Supplementary Figure S3**

**
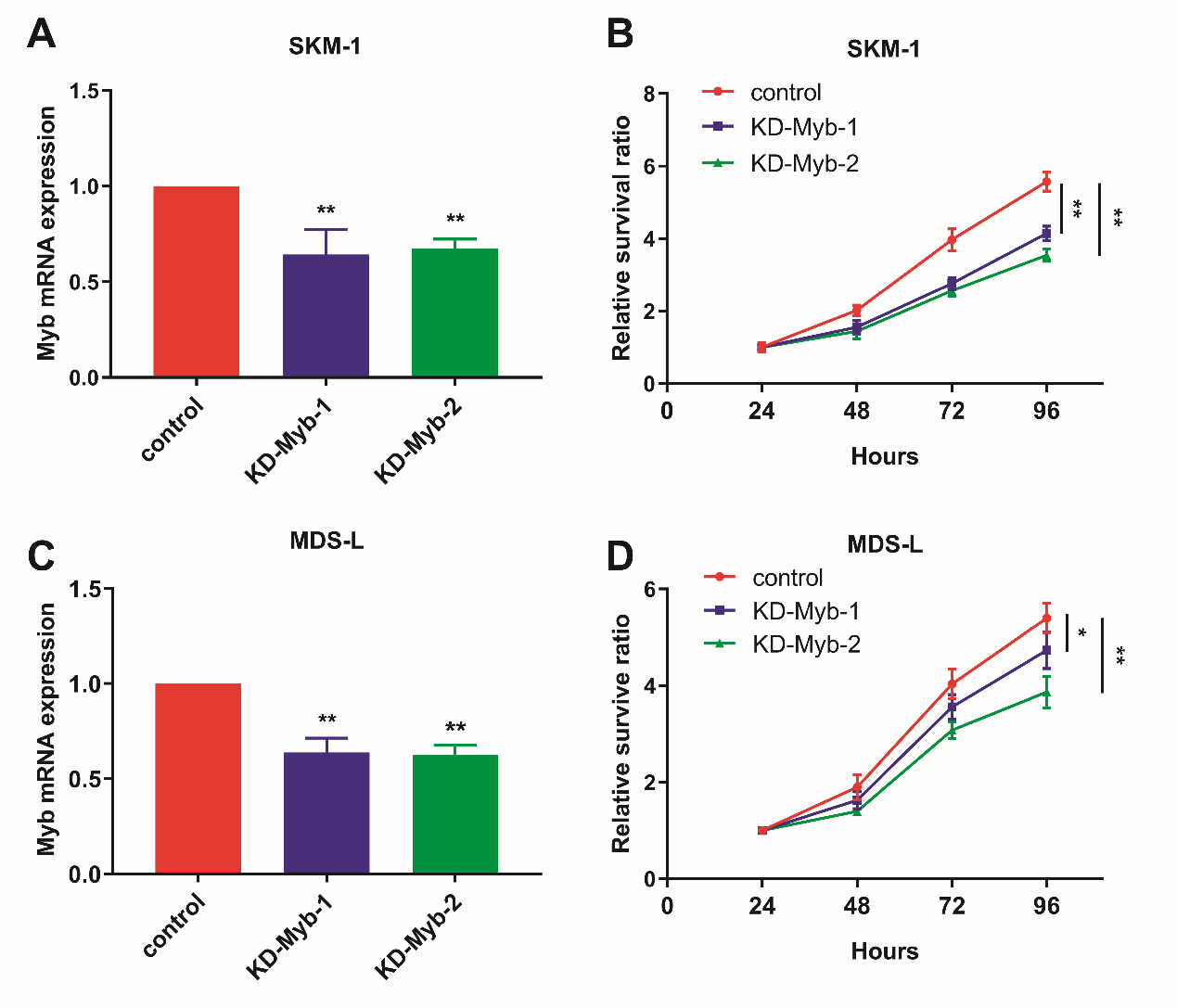
**

**Figure S3.** **Knockdown of Myb impaired the proliferation of MDS cells.**

**A** and **C** The expression of Myb was knocked down by two different siRNAs in both SKM-1 and MDS-L cells. **B** and **D** CCK-8 assays indicated that Myb knockdown suppressed SKM-1 and MDS-L cell proliferation. **p* < 0.05, ***p* < 0.01.

**Supplementary Figure S4**

**
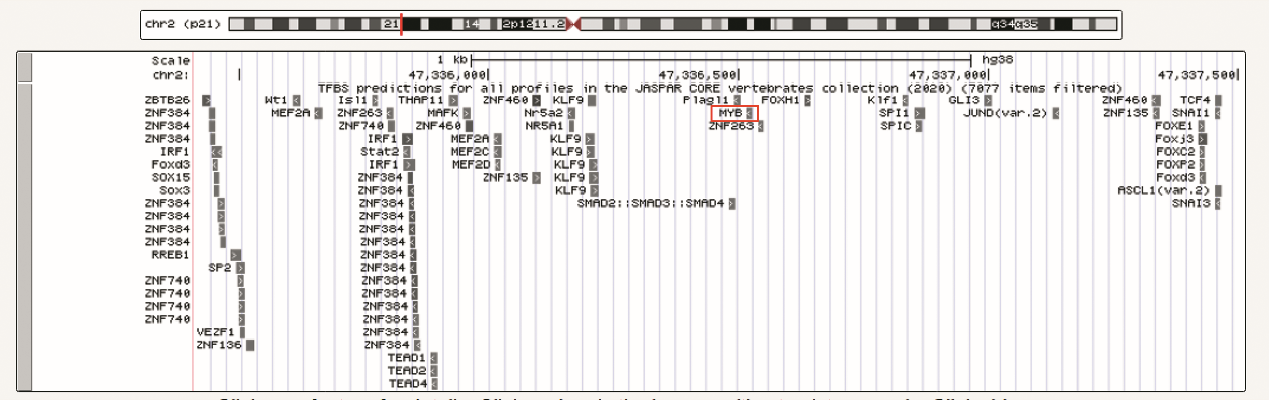
**

**Figure S4.** Analysis of the potential transcription factors in the upstream regulatory region of BC200 in UCSC Genome Browser. The red open squares indicated the putative Myb binding sites.

**Supplementary Figure S5**

**
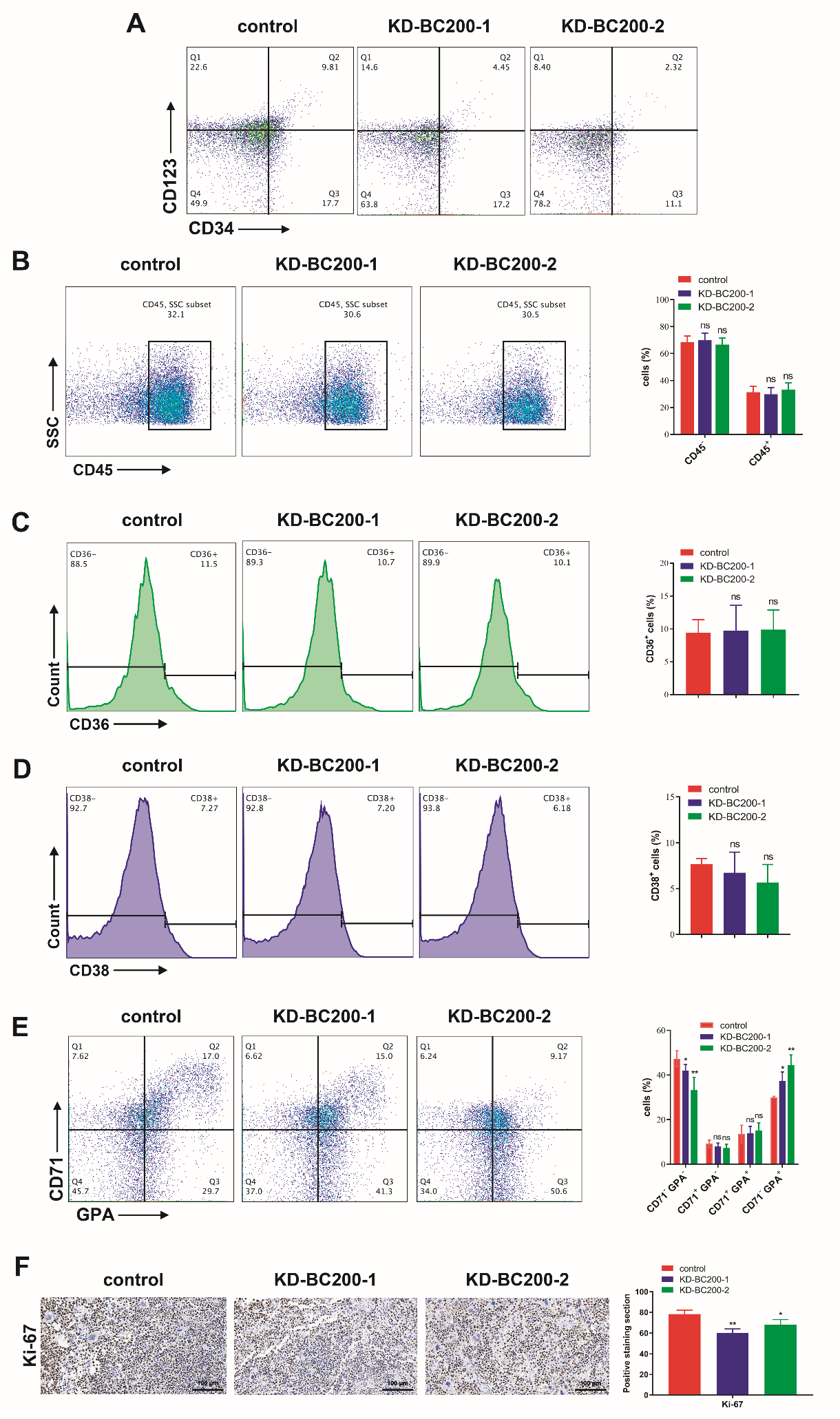
**

**Figure S5. Effects of knockdown BC200 in intravenous MDS mice.**

**A** Plot of CD123 vs CD34 expression of all BM cells of MDS or normal mice was analyzed by flow cytometry. **B** Proportions of CD45^-^ and CD45^+^ BM cells from MDS or normal mice was assessed by FACS. **C** and **D** Proportions of CD36^+^ or CD38^+^ BM cells from MDS or normal mice was assessed by FACS. **E** Plot of CD71 vs GPA expression of all BM cells of MDS or normal mice was analyzed. **F** The expression of Ki-67 of spleens collected from MDS or normal mice was detected by immunostaining. **p* < 0.05, ***p* < 0.01, ns, not significant.

**Supplementary Figure S6**


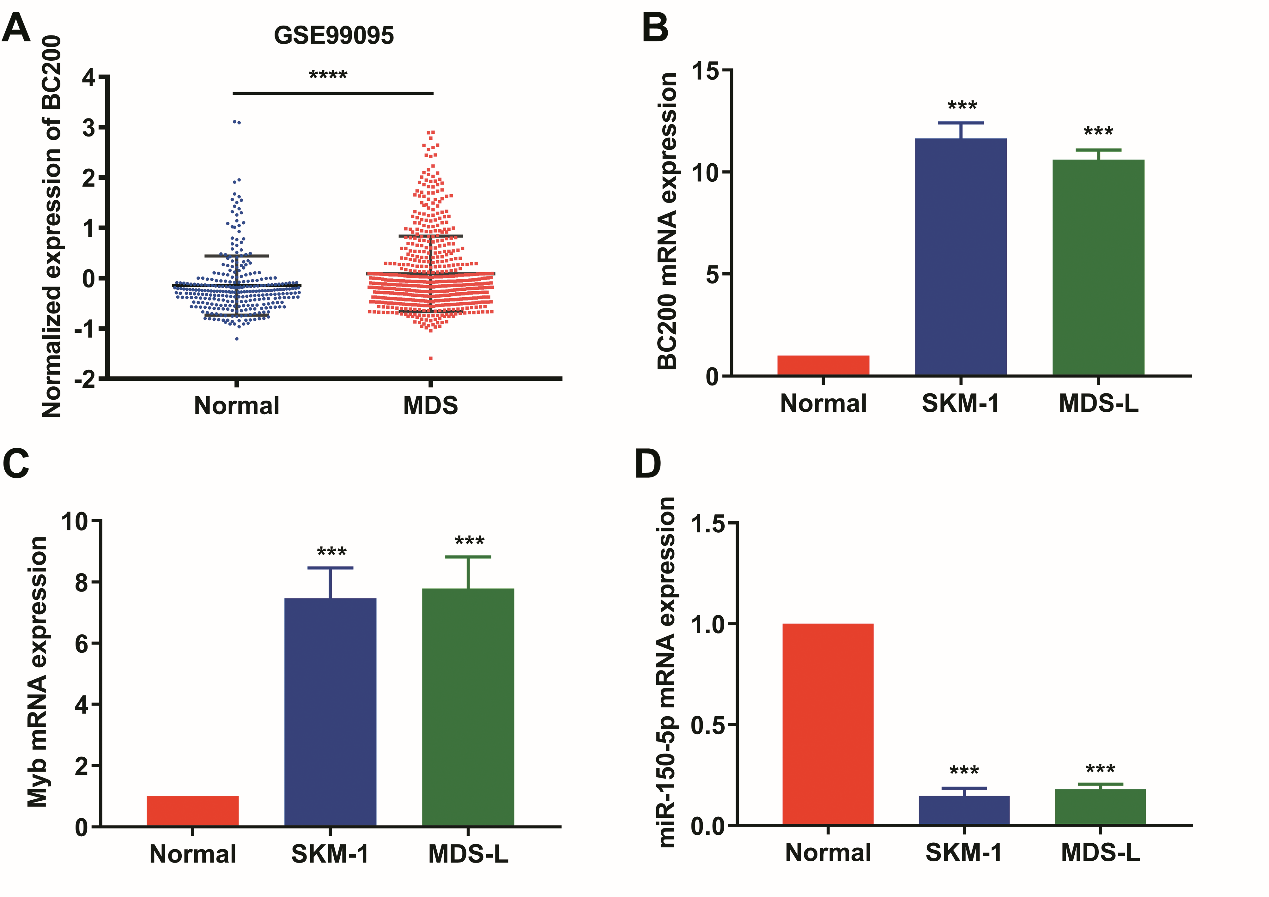


**Figure S6. The expression of BC200, miR-150-5p and Myb in MDS cells.**

**A** Normalized expression data of BC200 in human normal samples and MDS samples was obtained from the GEO database (GSE99095). **B-D** BC200, Myb and miR-150-5p expression in MDS cell lines (MSD-L and SKM-1) compared with normal human BMMCs. ****p* < 0.001, *****p* < 0.0001.

**Supplementary Figure S7**

**
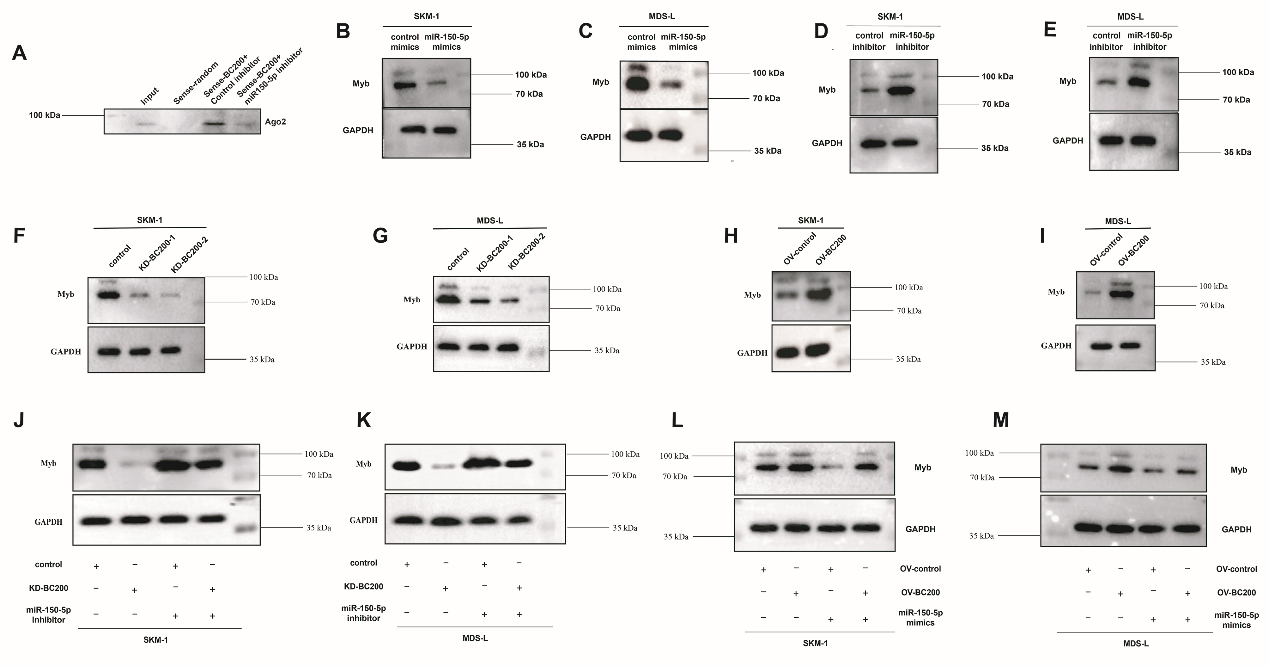
**

**Figure S7.** **Western blots with molecular weight markers**

**Supplementary Table S1. Sequences for gene knockdown used in this study.**

| **Gene name** | **Sequence** |
| --- | --- |
| si-BC200-1 | 5'-CGUAACUUCCCUCAAAGCAACAACC-3' |
| si-BC200-2 | 5'-GUAACUUCCCUCAAAGCAATT-3' |
| si-Myb-1 | 5'-GGACGAACUGAUAAUGCUATT-3' |
| si-Myb-2 | 5'-GAAAUACGGUCCGAAACGUTT-3' |

**Supplementary Table S2. qRT-PCR primers used in this study.**

| **Gene name** | **Forward Primer** | **Reverse Primer** |
| --- | --- | --- |
| BC200 | 5'-ATAGCTTGAGCCCAGGAGTT-3' | 5'-GCTTTGAGGGAAGTTACGCTTAT-3' |
| Myb | 5'-GGCGAGCCCCTTGCA-3' | 5'-CTCCTCCATCTTTCCACAGGAT-3' |
| miR-150-5p | 5'-AGTGCTGTCTCCCAACCCTT-3' | 5'-TATGGTTGTTCACGACTCCTTCAC-3' |
| miR-181b-5p | 5'-GCCGAACATTCATTGCTG-3' | 5'-TATGGTTCTTCACGACTCCTTCAC-3' |
| miR-590-3p | 5'-TCGCCCCTAATTTTATGTATAAGC‐3' | 5'-TATGGTTGTTCACGACTCCTTCAC-3' |
| U6 | 5'-CGCTTCGGCAGCACATATAC-3' | 5'-TTCACGAATTTGCGTGTCATC-3' |
| GAPDH | 5'-TGGTATCGTGGAAGGACTC-3' | 5'-AGTAGAGGCAGGGATGATG-3' |

**Supplementary Table S3. ChIP-qPCR primers used in this study.**

| **Gene name** | **Forward Primer** | **Reverse Primer** |
| --- | --- | --- |
| ChIP-BC200 promoter site 1 | 5'-CACCTCTCTTGTGGAGCCCT-3' | 5'-TTGTCCTGCCTGAGCCAGAG-3' |
| ChIP-BC200 promoter site 2 | 5'-AAACATCTTGGCTGGGCACG-3' | 5'-TCTCCTGACCTTGTGACCCG-3' |
| ChIP-BC200 promoter site NC | 5'-CAGAGCCAAAAGACACTCAAGGA-3' | 5'-TCAGGACATGAGGTGGGAGATAA-3' |
